# NaF PET assessment of bone metabolic changes around the femoral canal by intramedullary femoral alignment technique in total knee arthroplasty

**DOI:** 10.1002/ccr3.2187

**Published:** 2019-05-10

**Authors:** Soichiro Kaneko, Masakazu Kanetaka, Kei Wagatsuma, Kenji Ishii, Yorito Anamizu, Fumiaki Tokimura, Tsuyoshi Miyazaki

**Affiliations:** ^1^ Department of Orthopaedic Surgery Tokyo Metropolitan Geriatric Hospital and Institute of Gerontology Tokyo Japan; ^2^ Research Team for Neuroimaging Tokyo Metropolitan Geriatric Hospital and Institute of Gerontology Tokyo Japan

**Keywords:** extramedullary, intramedullary, NaF, PET, total knee arthroplasty

## Abstract

We used the NaF PET scan to assess osteometabolic changes around the distal half of the femoral canal by intramedullary (IM) drill for femoral IM guiding rod insertion in total knee arthroplasty. Gentle IM rod insertion and focused attention can minimize surgical stress‐induced biological reaction of the femoral IM canal.

## 1 INTRODUCTION

The recently developed ^18^F‐sodium fluoride ([^18^F] NaF) bone positron emission tomography (PET) is a more accurate diagnostic tool for specific bone disorders with the potential to replace conventional bone scintigraphy. Most of the NaF transported to the bone is retained only after a single pass of blood, making it a suitable radiopharmaceutical agent for the assessment of subtle changes in bone metabolic activity.

Total joint arthroplasty is a surgical procedure in which parts of an arthritic or damaged joint are removed and replaced with a metal, plastic, or ceramic device called prosthesis. The prosthesis is designed to replicate the movement of a normal, healthy joint. Total knee arthroplasty (TKA) and total hip arthroplasty (THA) are the most commonly performed joint replacement procedures, although replacement surgery can be performed on other joints as well, including the shoulder, elbow, wrist, and ankle. In addition to the improvement of the design and material properties of prostheses, advancements in surgical techniques, such as minimally invasive and muscle‐sparing approaches, have greatly increased the effectiveness of this technique. A recent study reported that TKA is one of the most successful procedures in medical practice, and the literature contains a number of reports describing prosthesis survival rates of more than 90% at the 30‐year follow‐up examination.^1^

Recently, we reported both visual and quantitative evaluations of the peri‐implant bone metabolic activity at the interface using [^18^F] NaF bone PET scans.^2^ After arthroplasty, bone metabolic activity increased in the first 7 days, with the highest level on postoperative days (POD) 7‐60 and returned to baseline level thereafter. In this study, we assessed bone metabolic changes around the distal half of the femoral canal using intramedullary (IM) drill for femoral IM rod insertion in TKA.

## 2 CASE PRESENTATION

### 2.1 Case 1

**Figure 1:**
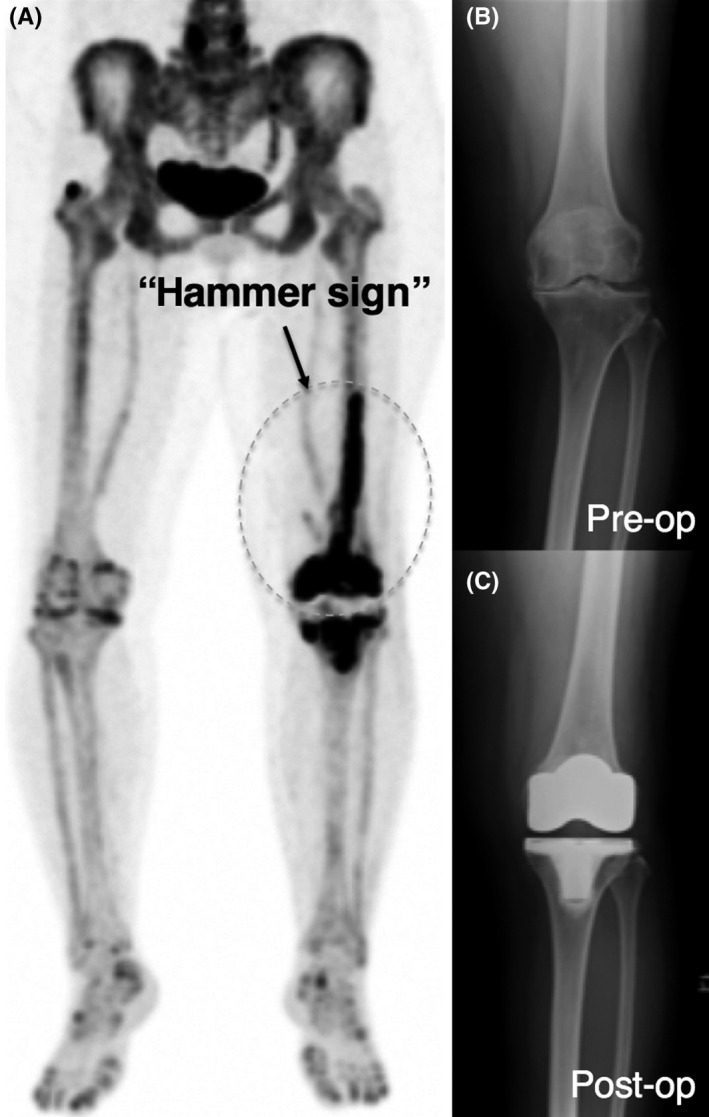
A 76‐year‐old woman was treated with a left TKA for osteoarthritis of the knee. Femoral and tibial cuts were achieved with preoperative planning using intramedullary femur jig and extramedullary tibial jig, respectively. (A) ^18^F‐sodium fluoride ([^18^F] NaF) bone positron emission tomography images, performed 2 wk after implantation, showed high bone metabolic activity at the distal half of the left femur in addition to the bone‐prosthesis interface called “hammer sign” (arrow). (B and C) X‐ray examination before and after joint arthroplasty

A 76‐year‐old woman was treated with a left TKA for osteoarthritis of the knee. Femoral and tibial cuts were achieved with preoperative planning using IM femur jig and extramedullary tibial jig, respectively. We visually analyzed and evaluated the tracer distribution around the TKA site 2 weeks after implantation. Interestingly, we recognized the “hammer sign,” which is hammer‐like increased signal intensity at the distal femur (high bone metabolic activity at the distal half of the right femur in addition to the bone‐prosthesis interface; Figure 1). We believe that the area with “hammer sign” is correlated with the surgical stress of the IM femoral canal, probably due to the IM drill for insertion of the femoral IM guiding rod for an appropriate femoral component positioning.

### 2.2 Case 2

**Figure 2:**
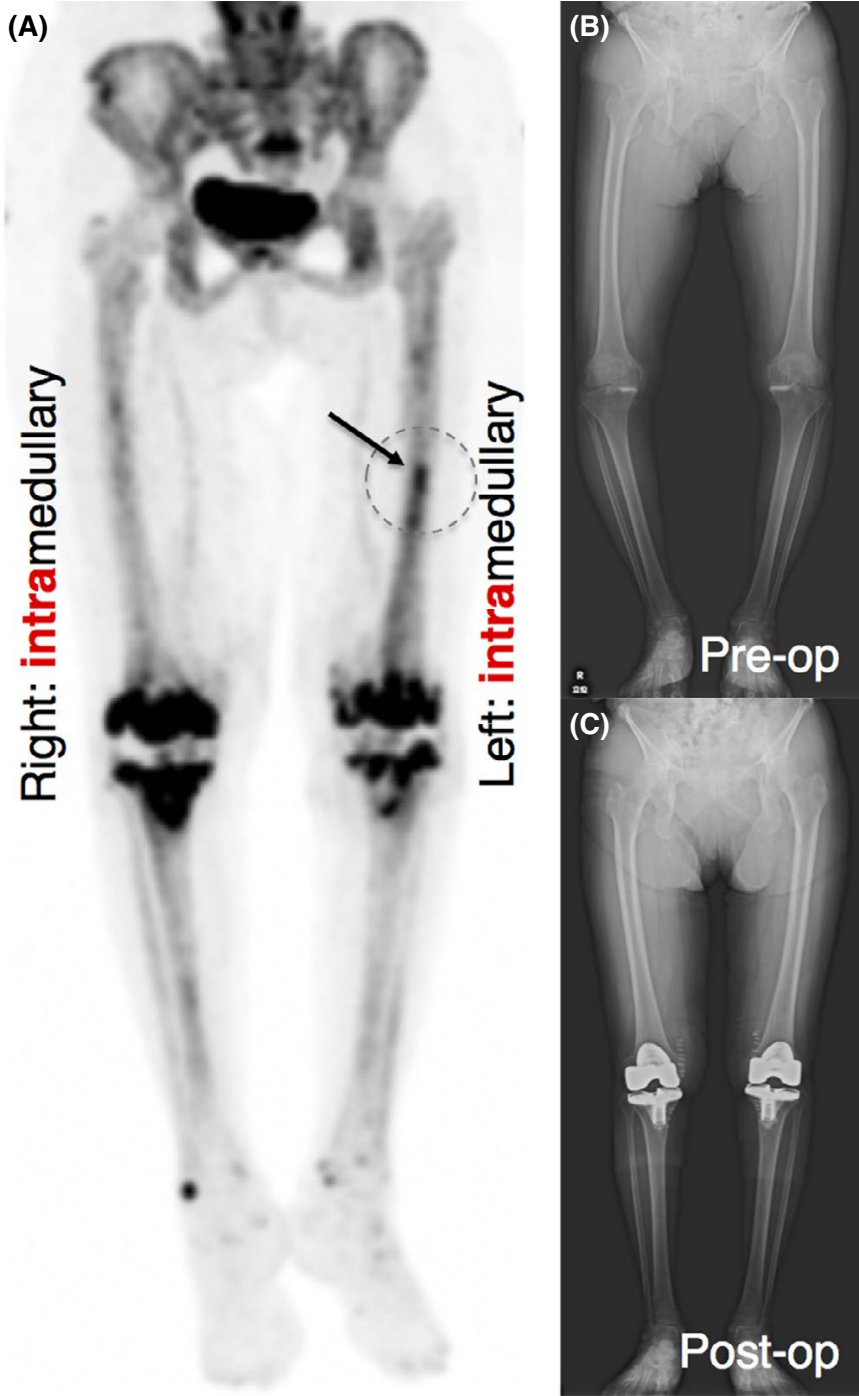
An 86‐year‐old woman was treated with simultaneous bilateral TKA for knee osteoarthritis. For bilateral replacement, femoral and tibial cuts were achieved with intramedullary femur jig and extramedullary tibial jig, respectively. Femoral intramedullary rods were slowly inserted with scrupulous care. (A) Postoperative ^18^F‐sodium fluoride ([^18^F] NaF) bone positron emission tomography showed that upregulation of the tracer uptake at the distal half of the right femur almost completely disappeared, although a slight bone metabolic activity at middle third of the left femur (arrow) was detected. (B and C) X‐ray examination before and after joint arthroplasty

An 86‐year‐old woman was treated with simultaneous bilateral TKA for knee osteoarthritis. For bilateral replacement, femoral and tibial cuts were achieved with IM femur jig and extramedullary tibial jig, respectively. During the surgery, we carefully reamed the entrance point and gently inserted a femoral IM rod, with the central axis of the distal femur as the ideal entry point. Unexpectedly, postoperative NaF PET imaging on POD 14 (Figure 2) demonstrated that the intensity of radiotracer uptake of the left femur was dramatically reduced without a typical “hammer” configuration, although a slight bone metabolic activity was detected at the middle third of the left femur. More interestingly, there was no upregulation of the NaF uptake in the right femur (Figure 2).

### 2.3 Case 3

**Figure 3:**
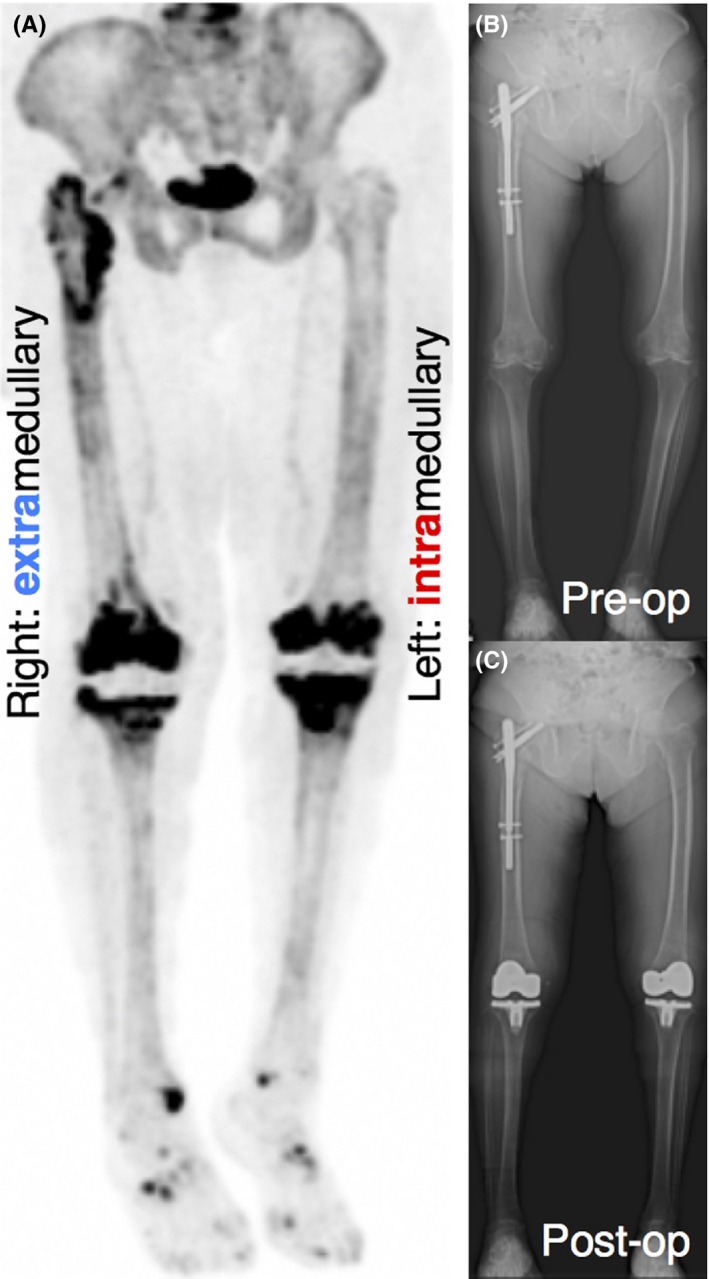
An 81‐year‐old woman, who suffered from right femoral intertrochanteric fracture 5 mo earlier, was treated with simultaneous bilateral TKA for knee osteoarthritis. (A) Bilateral TKA was performed with the extramedullary guide for the right and the IM alignment system for the left femur. The “hammer sign” was not observed in not only the right femur but also the left, indicating that slow insertion of an intramedullary rod with scrupulous care can reduce the damage to the femoral canal to the same level as that in extramedullary femoral alignment guide system. (B and C) X‐ray examination before and after joint arthroplasty

An 81‐year‐old woman, who suffered from right femoral intertrochanteric fracture and had right hip open reduction and internal fixation (ORIF) with gamma nail 5 months earlier, was treated with simultaneous bilateral TKA for knee osteoarthritis. Simultaneous bilateral TKA was performed with the extramedullary guide for the right femur and the IM alignment system for the left femur. KneeAlign 2, a simple palm‐sized navigation device, was used for extramedullary femoral alignment.^3^ As shown in Figure 3, NaF PET image on POD 14 showed no significant difference in signal intensity between the right and left distal femurs, although the high intensity around the right hip may be triggered by a previous trauma and surgery. This indicates that gentle IM rod insertion with scrupulous care could minimize breaching of the femoral canal as well as the extramedullary femoral alignment guide system with KneeAlign 2.

## 3 DISCUSSION

In this study, we performed visual evaluation of bone metabolic changes around the distal half of the femur using IM drill for femoral IM rod insertion in TKA. We found that gentle IM rod insertion and focused attention can minimize the intensity of radiotracer uptake at the femoral canal.

Recent advances in TKA have focused on the reduction of damage during the procedure.^4^, ^5^, ^6^ One of the most invasive parts of TKA is the violation of the IM femoral canal by placement of an IM alignment rod. The use of an IM guide for the femur can result in various complications such as blood loss, postoperative hypoxia, intraoperative fractures, and fat embolism.^4^, ^7^, ^8^ We speculate that the area with the “hammer sign” correlated with the breaching of the IM femoral canal, probably due to the IM drill for the insertion of the femoral IM guiding rod for an appropriate femoral component positioning. To prevent unnecessary complications, we need to carefully ream the entrance point and gently insert a femoral IM rod, with the central axis of the distal femur as the ideal entry point. Briefly, the entrance hole, approximately 1 cm anterior to the origin of the posterior cruciate ligament, should be parallel to the femoral shaft in both anteroposterior and lateral projections and enlarged to reduce unnecessary mechanical stress during subsequent rod insertion. Then, the direction for the AP view is determined using the anterosuperior iliac spine as an intraoperative landmark. Furthermore, the direction for the lateral view is determined by palpating the femoral shaft, although its assessment was difficult owing to the large soft tissue cover. Finally, the femoral guiding rod was carefully compressed to avoid the contact of the tip of the rod with the cortex of the femur in all directions.

In general, the uptake pattern (hammer‐like increased signal intensity at distal femur) found in Case 1 is considered to be a violation of the IM femoral canal leading to complications such as increased blood loss. From another point of view, however, an increased tracer uptake might represent the improvement of the bone metabolic conditions inside the bone marrow cavity. Further investigation of the biological reaction of bone turnover after surgical stress in patients will provide new insights into the molecular mechanisms regulating bone remodeling.

## 4 CONCLUSION

In this study, we successfully used NaF PET scans for the evaluation of bone metabolic changes around the distal femur using IM drill for femoral IM rod insertion in TKA, and we found that our surgical procedure controlled the osteometabolic activity at the distal femoral canal and that gentle insertion of an IM rod with scrupulous care could reduce the tracer uptake to the femoral canal to the same level as that in extramedullary femoral alignment guide system. Appropriate tissue and equipment handling with close attention to details can help minimize the effect on the bone marrow.

## INFORMED CONSENT

Informed consent has been obtained for the publication of the images.

## CONFLICT OF INTEREST

The authors have declared that no conflict of interest exists.

## AUTHOR CONTRIBUTIONS

All authors: made substantial contribution to the preparation of this manuscript. SK: wrote the preliminary manuscript. MK, YA, and FT: completed the literature search and found the relevant articles for the discussion. KW and KI: provided PET images. TM: supervised all the work.
